# A Middle-Aged Man Presenting With Progressive Heart Failure, Myopathy, and Monoclonal Gammopathy of Uncertain Significance

**DOI:** 10.1016/j.jaccas.2020.02.024

**Published:** 2020-04-08

**Authors:** Kaspar Broch, Trine Popperud, Einar Gude, Yngvar Fløisand, Ellen-Ann Antal, Gerhard Bosse, Christoffer Jonsrud, Terje Hegard, Silje Skaara, Ahmed Elsais

**Affiliations:** a Department of Cardiology, Oslo University Hospital Rikshospitalet, Oslo, Norway; b Department of Neurology, Oslo University Hospital Rikshospitalet, Oslo, Norway; c Department of Hematology, Oslo University Hospital Rikshospitalet, Oslo, Norway; d Department of Pathology, Oslo University Hospital Rikshospitalet, Oslo, Norway; e Department of Radiology and Nuclear Medicine, Oslo University Hospital Rikshospitalet, Oslo, Norway; f Department of Medical Genetics, University Hospital of North Norway, Tromsø, Norway; g Department of Medicine, Telemark Hospital Trust, Notodden Hospital, Notodden, Norway; h Department of Neurology, Telemark Hospital Trust, Skien, Norway

**Keywords:** cardiomyopathy, chronic heart failure, echocardiography, imaging, right-sided catheterization, MGUS, monoclonal gammopathy of uncertain significance, SLONM, sporadic late-onset nemaline myopathy

## Abstract

A 48-year-old man presented with rapidly progressive heart failure and monoclonal gammopathy of uncertain significance. No specific cause was detected on endomyocardial biopsy. As the heart failure worsened, he also developed progressive skeletal myopathy. This provided the clue to the diagnosis, and cardiac function recovered rapidly with cause-directed therapy. (**Level of Difficulty: Intermediate.**)

## Graphical abstract

**Figure:**
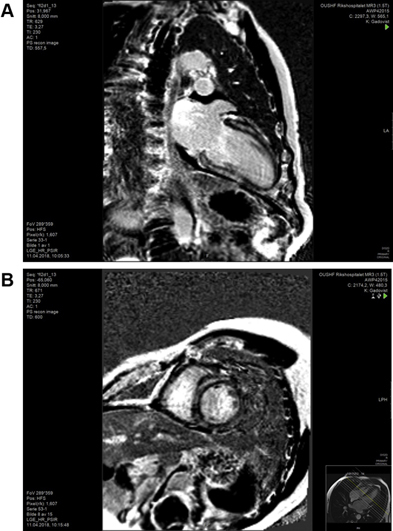

## History of Presentation and Past Medical History

A 48-year-old man who had been diagnosed with type 2 diabetes a year previously, presented with progressive dyspnea. There was no family history of heart disease. On first admission, he was dyspneic during speaking. There was no peripheral edema. On lung auscultation, bibasilar crackles were heard. There was no cardiac murmur. His left ventricle had mild to moderate systolic dysfunction and diastolic impairment. Vital parameters are provided in Table 1. After temporary symptomatic relief with the introduction of guideline-directed drug therapy for heart failure, his dyspnea worsened, and approximately 1 year after symptom onset, the patient was once again in New York Heart Association functional class III.

**Learning Objectives**

• To understand that SLONM is a rare, potentially treatable cause of heart failure.
• To know how to diagnose and treat SLONM associated with monoclonal gammopathy and heart failure.

**Table 1:** Clinical and Hemodynamic Variables

	On First Admission	18 Months Later	Before Start of Specific Therapy∗	2 Months After the Initiation of Therapy
NYHA functional class	IV	III	IV	I
Blood pressure, mm Hg	156/116	145/100	117/96	115/85
Heart rate, beats/min	106	90	110	74
NT-proBNP, pg/l	3,061	3,540	23,246	1,023
LV internal diameter, cm†	5.6	5.8	5.6	5.8
LV septum thickness, cm†	1.0	0.9	1.1	1.1
LVEF, %	40	40‡	18	54
Right heart catheterization				
Right atrial pressure, mm Hg		7	18	
MPAP, mm Hg		38	47	
PCWP, mm Hg		24	34	
Cardiac output, l/min		5.9	3.8	6.3†
Mixed venous oxygen saturation, %		63	43	

LV = left ventricle; LVEF = left ventricular ejection fraction; MPAP = mean pulmonary arterial pressure; NT-proBNP = N-terminal pro–B-type natriuretic peptide; NYHA = New York Heart Association; PCWP = pulmonary capillary wedge pressure.

∗ Bortezomib, lenalidomide, and dexamethasone.

† Measured by echocardiography.

‡ Measured by cardiac magnetic resonance imaging.

## Investigations

**Figure 1:**
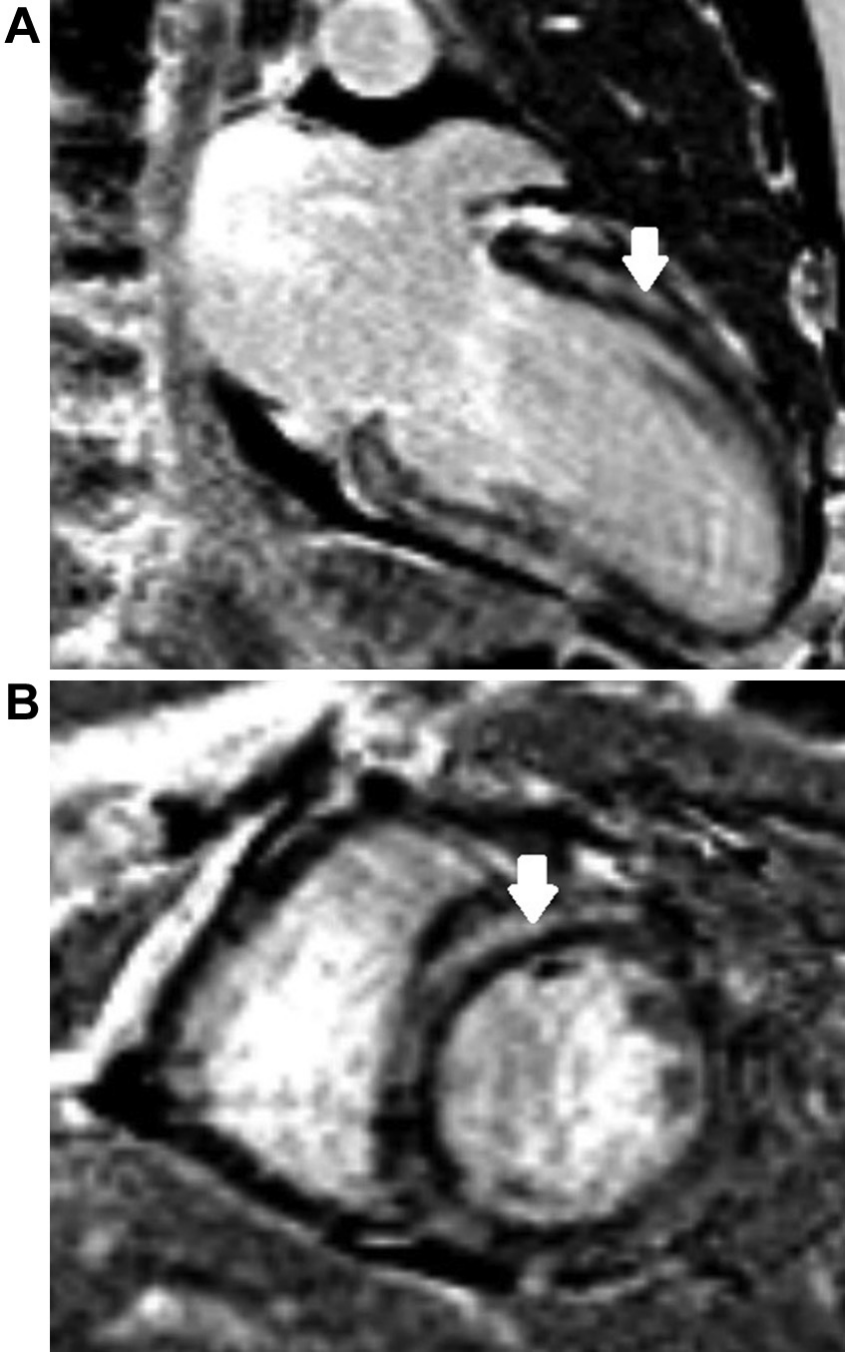
CMR Imaging Two-chamber long-axis **(A)** and short-axis **(B)** views showing midwall and subepicardial late gadolinium enhancement **(arrows)**.

**Figure 2:**
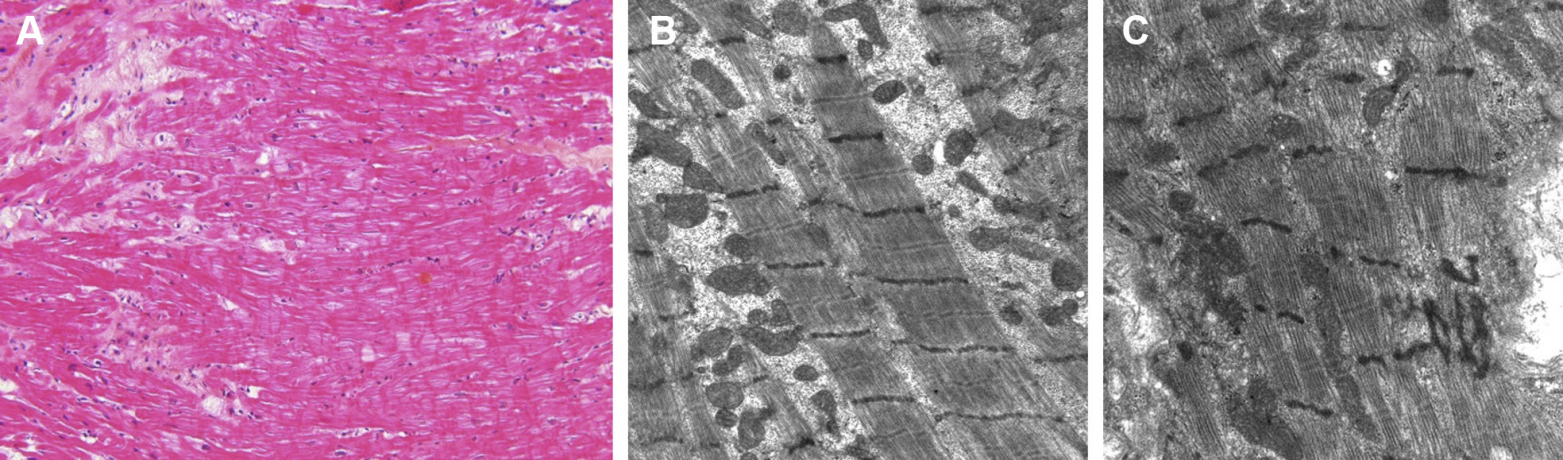
Endomyocardial Biopsy **(A)** Light micrograph from a hematoxylin and eosin–stained section. There is focal loss of muscle elements and repair by fibrosis **(upper left)**. **(B,C)** Electron micrograph showing normal myocytes **(B)** and focal Z-disc streaming **(C)**. No sarcoplasmic rods were observed.

Coronary angiogram revealed nonobstructive coronary artery disease. Echocardiography showed moderately impaired left ventricular systolic function, signs of elevated left ventricular filling pressure, but no clinically significant valvular disease (Videos 1 and 2). Right heart catheterization revealed post-capillary pulmonary hypertension (Table 1). Cardiac magnetic resonance imaging confirmed mild left ventricular dilation and mild systolic dysfunction. There were pathological values on native T_1_ mapping and extensive late gadolinium enhancement in the midwall and subepicardial layers of the left ventricle. The enhancement pattern could suggest inflammation/myocarditis, but due to the extent of these findings infiltrative heart disease was discussed even though there was no myocardial hypertrophy (Figure 1). Cardiac biopsy demonstrated mild fibrosis and myocyte hypertrophy, but no signs of amyloid or other causes of infiltrative disease (Figure 2). Supplementary work-up revealed an immunoglobulin G lambda monoclonal band in the serum (1 g/l). The proportion of plasma cells in the bone marrow was 1% to 3%, which is consistent with a diagnosis of monoclonal gammopathy of uncertain significance (MGUS).

**Online Video 1:** Color Doppler Echocardiogram 18 Months After Symptom Onset

Apical, long-axis view of the heart showing mild dilation of the left ventricle, moderately reduced left ventricular function, and a preserved valvular apparatus.

**Online Video 2:** Gray-scale Echocardiogram 18 Months After Symptom Onset

Parasternal long-axis view showing modest hypertrophy, moderate systolic dysfunction.

It was noted that the patient had an abnormal gait, and he complained of having trouble walking fully upright, but magnetic resonance imaging showed no pathology in the spinal cord, spinal nerve roots, or spine. On neurological examination, we observed fasciculations, proximal muscular atrophy, and axial and proximal muscle weakness. The sensory examination was normal. Tendon reflexes were brisk and the plantar response normal. Blood levels of creatine kinase were normal. The cerebrospinal fluid was normal apart from a slightly elevated protein level. A computed tomography scan revealed no thoracic, abdominal, or pelvic malignancy. The electromyogram and muscle biopsies showed signs of myopathy.

## Differential Diagnosis

**Figure 3:**
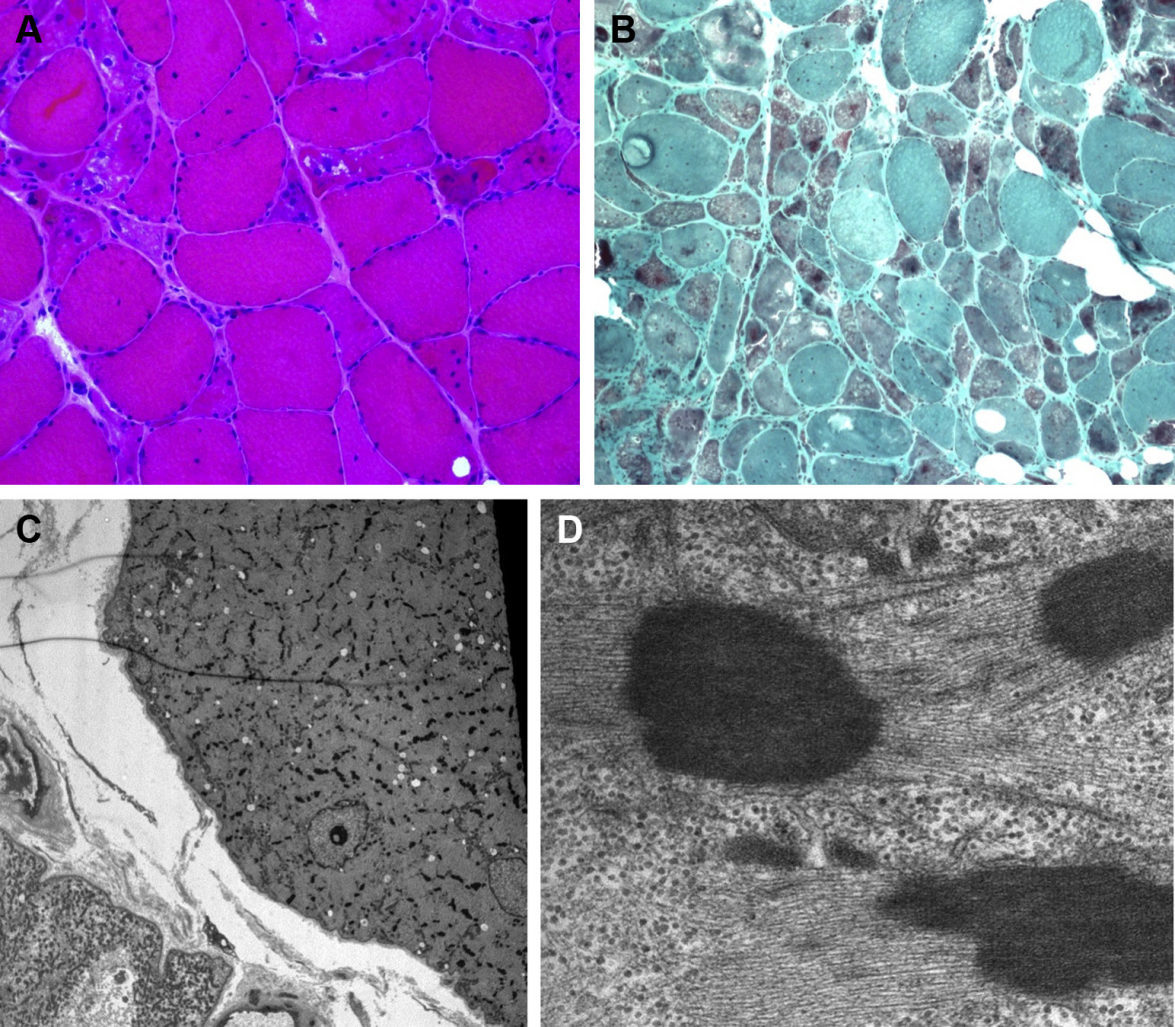
Muscle Biopsy (Vastus Lateralis) **(A)** Light micrograph from a hematoxylin and eosin–stained section showing chronic myopathy. **(B)** Gomori trichrome. There is extensive deposition of cytoplasmic granular material: nemaline rods. **(C, D)** Electron microscopy. Multiple sarcoplasmic nemaline rods can be observed.

The combination of myopathy and heart failure was suggestive of inherited muscle disease. We performed a genetic work-up that included a large panel of genes known to cause myopathy and cardiomyopathy (see the overview of genes sequenced in the Supplemental Appendix), without discovering a genetic cause of the patient’s disease. Monoclonal gammopathy of uncertain significance can cause cardiac amyloid light-chain amyloidosis through the deposition of free light chains. Due to high suspicion of infiltrative disease, we repeated endomyocardial biopsy, but again, there was no sign of amyloid. On renewed examination of deltoid and vastus skeletal muscle biopsies, however, we found abundant sarcoplasmic nemaline rods consistent with sporadic late-onset nemaline myopathy (SLONM) (Figure 3). On first look, these inclusions had not been recognized for what they were.

## Management

Approximately 2 years after first being diagnosed with heart failure, the patient was in New York Heart Association functional class IV with dyspnea at rest despite optimal pharmacological treatment for heart failure. His hemodynamics were severely compromised (Table 1, Videos 3 and 4). He had pronounced axial and proximal muscle weakness and atrophy, dysphagia, and dropped head. He had difficulties walking unaided and rising from a chair. We regarded MGUS-associated SLONM as the likely cause of his myopathy and heart failure but considered him too ill to tolerate high-dose chemotherapy with autologous stem-cell support. Instead, we decided to initiate front-line therapy for MGUS to provide disease control. Shortly before admission for the initiation of therapy for MGUS, the patient developed atrial flutter with an average ventricular rate of approximately 110 beats/min. He received ablation therapy for atrial flutter, after which we begun treatment as in plasma cell disease with bortezomib, lenalidomide, and dexamethasone. The monoclonal component could not be detected after start of treatment.

**Online Video 3:** Color Doppler Echocardiogram Acquired Prior to the Initiation of Therapy Directed at the Plasma Cell Clone

Apical long-axis view of the heart showing mild dilation of the left ventricle, severely reduced left ventricular function, and a preserved valvular apparatus.

**Online Video 4:** Gray-scale Echocardiogram Acquired Prior to the Initiation of Therapy Directed at the Plasma Cell Clone

Parasternal long-axis view showing severe systolic dysfunction.

## Follow-Up

After the initiation of therapy, the patient rapidly recovered. Two months later, his left ventricular function had normalized (Videos 5 and 6), and he was in New York Heart Association functional class I (Table 1). On physical testing, he had improved muscle strength, but persistent weakness of the proximal and axial muscles. He was deemed fit to tolerate high-dose chemotherapy with autologous stem-cell support, which we recently performed without severe side effects.

**Online Video 5:** Color Doppler Echocardiogram Acquired Approximately 2 Months After the Initiation of Cause-directed Therapy

Apical long-axis view of the heart showing near normalization of left ventricular function.

**Online Video 6:** Gray-scale Echocardiogram Acquired Approximately 2 Months After the Initiation of Cause-directed Therapy

Parasternal long-axis view showing near normalization of left ventricular function.

## Discussion

Skeletal myopathy and cardiomyopathy coexist in several hereditary disorders including mitochondriopathies, storage diseases, and diseases caused by mutations in genes encoding structural proteins (1). A family history of myopathy or cardiomyopathy and either early-onset or slowly progressive disease supports the diagnosis of hereditary myopathy with cardiomyopathy. In this case, we could not find a genetic cause, and the coexistence of MGUS eventually led us to look for a different explanation. MGUS is a premalignant disease caused by the monoclonal expansion of a plasma cell. It carries a 1% yearly risk of progressing to multiple myeloma, lymphoma, amyloid light-chain amyloidosis, macroglobulinemia, lymphocytic leukemia, or plasmacytoma (2). MGUS is known to cause cardiac amyloid light-chain amyloidosis through the deposition of free light chains (3), but it is also associated with the rare myopathy SLONM (4).

Nemaline myopathy is usually an early-onset, inherited disease caused by mutations in genes encoding myocyte structural proteins. The rare sporadic late-onset variety has been associated with MGUS. In a few cases, concomitant heart failure has been reported (4). In SLONM, the MGUS presumably causes myopathy through interaction between the monoclonal immunoglobulins and the sarcomeric proteins of the myocytes (4). Presumably, the same mechanism is responsible for the heart failure, but notably, we did not find nemaline bodies on endomyocardial biopsy.

In case series, successful treatment of MGUS has led to recovery from the myopathy (4,5), and in 2 single cases, simultaneous recovery from heart failure has been reported (6,7). In the latter cases, SLONM with MGUS preceded the onset of heart failure. In our patient, on the other hand, heart failure preceded the onset of symptomatic myopathy by several months. SLONM with MGUS should therefore be considered in the diagnostic evaluation not only in progressive myopathy, but also in unexplained heart failure. Importantly, skeletal muscle, biopsy, but not endomyocardial biopsy, was diagnostic in our case. By the time treatment for MGUS was initiated, our patient had severe, end-stage heart failure, which nevertheless improved rapidly. Notably, the left ventricular function recovered more rapidly than skeletal muscle function, suggesting that the pathophysiologic mechanism of heart failure in SLONM with MGUS is different from that in the skeletal muscles. This is supported by the fact that nemaline bodies were not detected in 2 separate sets of endomyocardial biopsies in our patient.

## Conclusions

Our case highlights a rare cause of heart failure amenable to cause-directed therapy. SLONM is a disease that primarily affects skeletal muscle, but can also cause heart failure. Our case illustrates the potential for recovery from severe heart failure with treatment directed at plasma cells in SLONM with MGUS.

## Appendix

Supplemental Appendix
