# Supplementary material for: A Middle-Aged Man Presenting With Progressive Heart Failure, Myopathy, and Monoclonal Gammopathy of Uncertain Significance
Source: JACC Case Rep. 2020 Apr 8;2(5):785–9. doi: 10.1016/j.jaccas.2020.02.024 (PMC8301701; doi:10.1016/j.jaccas.2020.02.024)
Supplement: Supplemental Appendix [file mmc1.docx]

<AH1>Overview of the Genes Sequenced in Our Search for a Hereditary Cause of Combined Myopathy and Cardiomyopathy

Three Norwegian accredited genetic laboratories used 3 different targeted next-generation sequencing panels with normal results:

- TruSight Cardio Sequencing Kit (Illumina, San Diego, California) with 174 genes.
- Bioinformatically targeting 195 myopathy associated genes in whole-exome based Nextera Rapid Capture Custom Kit (Illumina).
- Bioinformatically targeting 383 neuromuscular disorder associated genes in TruSight One.

Expanded Sequencing Panel (Illumina). Information on the specific genes in the 3 panels can be supplied on request.
